# A Model of the Intracellular Response of an Olfactory Neuron in *Caenorhabditis elegans* to Odor Stimulation

**DOI:** 10.1371/journal.pone.0042907

**Published:** 2012-08-23

**Authors:** Mamoru Usuyama, Chisato Ushida, Ryuzo Shingai

**Affiliations:** 1 Laboratory of Bioscience, Faculty of Engineering, Iwate University, Morioka, Japan; 2 The United Graduate School of Agricultural Sciences, Iwate University, Morioka, Japan; 3 Department of Biochemistry and Molecular Biology, Faculty of Agriculture and Life Science, Hirosaki University, Hirosaki, Japan; 4 RNA Research Center, Hirosaki University, Hirosaki, Japan; Harvard University, United States of America

## Abstract

We developed a mathematical model of a hypothetical neuronal signal transduction pathway to better understand olfactory perception in *Caenorhabditis elegans*. This worm has only three pairs of olfactory receptor neurons. Intracellular Ca^2+^ decreases in one pair of olfactory neurons in *C. elegans*, the AWC neurons, following application of an attractive odor and there is a transient increase in intracellular Ca^2+^ following removal of odor. The magnitude of this increase is positively correlated with the duration of odor stimulation. Additionally, this Ca^2+^ transient is induced by a cGMP second messenger system. We identified likely candidates for the signal transduction molecules functioning in this system based on available gene expression and physiological data from AWCs. Our model incorporated a G-protein-coupled odor receptor, a G-protein, a guanylate cyclase as the G-protein effector, and a single phosphodiesterase. Additionally, a cyclic-nucleotide-gated ion channel and a voltage-gated ion channel that mediated calcium influx were incorporated into the model. We posited that, upon odor stimulation, guanylate cyclase was suppressed by the G-protein and that, upon cessation of the stimulus, the G-protein–induced suppression ceased and cGMP synthesis was restored. A key element of our model was a Ca^2+^-dependent negative feedback loop that ensured that the calcium increases were transient. Two guanylate cyclase-activating proteins acted on the effector guanylate cyclase to negatively regulate cGMP signaling and the resulting calcium influx. Our model was able to closely replicate *in silico* three important features of the calcium dynamics of AWCs. Specifically, in our simulations, [Ca^2+^] increased rapidly and reached its peak within 10 s after the odor stimulus was removed, peak [Ca^2+^] increased with longer odor exposure, and [Ca^2+^] decreased during a second stimulus that closely followed an initial stimulus. However, application of random background signal (‘noise’) showed that certain components of the pathway were particularly sensitive to this noise.

## Introduction

*Caenorhabditis elegans* nematode worms exhibit well-characterized responses to defined external stimuli, and their nervous system is also well characterized. Therefore, this species is suitable for mathematical modeling of nerve and nervous system function. The nervous system of *C. elegans* comprises only 302 neurons; consequently, the role of a single neuron in the worm may be more important than that of any single neuron in the larger nervous systems of higher animals. Accurate physiological and signal-transduction-gene expression data from a neuron are essential for developing models of signal transduction in that neuron. Electrophysiological studies [1]–[3] are limited in this organism because the neurons are small; however, numerous recent studies have used genetically encoded calcium indicators for physiological analyses [4], [5]. Signal transduction in sensory neurons is relatively well characterized because of extensive genetic and behavioral analyses. The worm has only three pairs of olfactory receptor neurons. A pair of AWC neurons satisfy the abovementioned two requirements for mathematical modeling; these neurons sense attractive odors, including isoamyl alcohol, butanone, and benzaldehyde. AWC neurons exhibit a transient increase in intracellular calcium concentration ([Ca^2+^]) after the cessation of odor stimulus [6]. In vertebrate olfactory neurons, cAMP is increased by application, but not by removal, of stimuli [7]. In a model of an olfactory receptor neuron in frogs [8] that is based on electrophysiological data, a stimulus activates a receptor and a G-protein, which then activates adenylate cyclase, and, consequently, cAMP concentration increases. Binding of cAMP to cyclic nucleotide-gated (CNG) channels produces a calcium ion influx, which results in an excitation of membrane potential. However, in AWC neurons of *C. elegans*, cGMP is more likely to be the second messenger because CNG channels [9] and guanylate cyclase [10] are required for the response, although the odor transduction pathway in this neuron is not well characterized.

Here, we constructed a model of the odor transduction pathway in AWC neurons that can replicate *in silico* the [Ca^2+^] changes observed in AWC physiological experiments. We propose that, upon odor stimulation, guanylate cyclase is suppressed by a G-protein and that, upon cessation of the stimulus, the suppression ceases and cGMP synthesis is restored. Increases in cGMP concentration induce calcium ions influx via cGMP-sensitive CNG channels. Also the pathway includes feedback regulation of guanylate cyclase activity.

## Results

### Qualitative scheme of signal transduction

**Figure 1:**
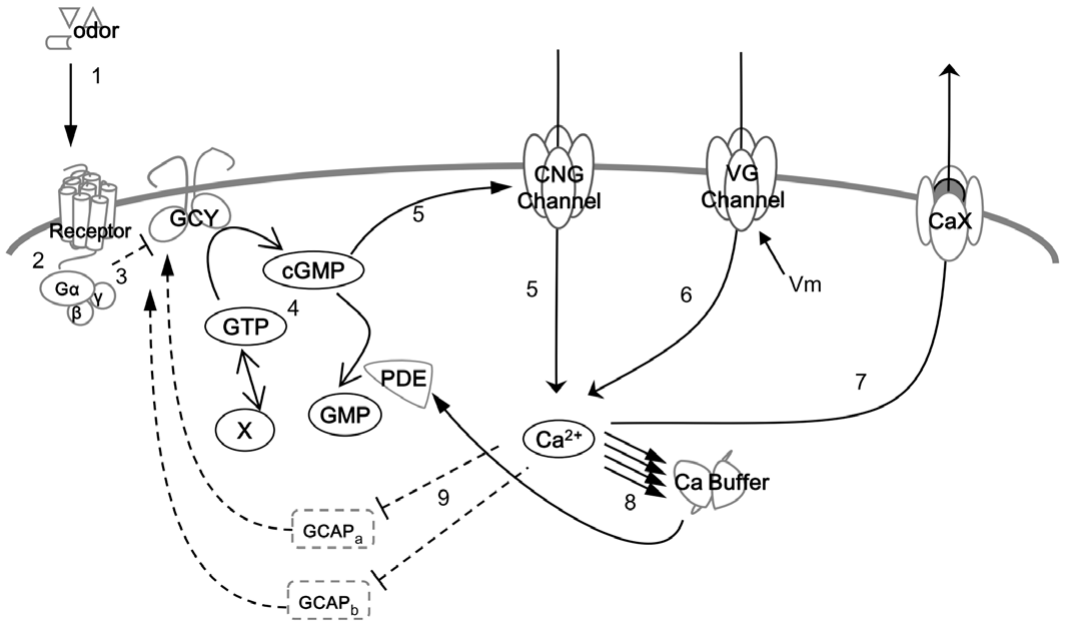
The hypothetical scheme of signal transduction. Reactions are classified into nine steps and numbered as reaction 1 thru 9. 1. Binding of an odor molecule to the receptor when odor stimulus is applied. Dissociation of an odor molecule from the receptor when odor stimulus is removed. 2. Activation of G-protein (Gα subunit) following the bind of odor molecule to the receptor. Inactivation of Gα subunit following an odor molecule dissociates from the receptor. 3. Inhibition of GCY by Gα. This process includes binding of GCY and Gα, GCY::GCAPa and Gα, and GCY::GCAPb and Gα. 4. Synthesis and decomposition of cGMP. These include cGMP synthesis by GCY, cGMP decomposition by PDE, and supply and removal of GTP by unknown process. 5. Binding of cGMP to the CNG channel, and changes in [Ca^2+^] and membrane potential via the CNG channel. 6. Changes in [Ca^2+^] and membrane potential via a voltage-gated channel (voltage-dependent calcium channel). 7. Change in [Ca^2+^] via a calcium extrusion mechanism (CaX). 8. Binding of calcium ions to the calcium buffer (CaM), and modulation of CaX and PDE. 9. Inactivation of GCAPs by calcium ions and feedback on GCY activity.

To obtain insight into the mechanisms that mediate changes in [Ca^2+^], we searched the literature and the *C. elegans* database (http://www.wormbase.org) to identify molecules that are reportedly expressed in *C. elegans* neurons. The physiological responses of AWC neurons to olfactory stimuli have been studied primarily using calcium imaging, and the fluorescent protein G-CaMP [6], [11]. [Ca^2+^] increases transiently after removal of odor stimulation, but the signal transduction mechanism remains unclear. We proposed a hypothetical scheme that could replicate this phenomenon *in silico*; we introduced likely candidate molecules into this scheme to represent components of this unknown mechanism. (Fig. 1).

#### Stimulus and reception

In our model, we theorized that the olfactory receptors were G-protein-coupled receptors. Following a pulse stimulus to the neuron, the receptor activated G-proteins such as ODR-3 (Gα subunit) [12], GPB-2 (Gβ subunit), and GPC-1 (Gγ subunit). The *odr-3* mutants are defective in chemotaxis to odorants sensed by AWC neurons [12]. The active G-proteins then activate effectors, which have not been identified.

#### Supposition for effectors

The effector of the Gα subunit has not been identified; therefore, we selected a likely candidate, guanylate cyclase (GCY), as the hypothetical effector. The [Ca^2+^] changes in AWCs resemble the well-characterized [Ca^2+^] responses that occur in the photoreceptor cell of vertebrate retina. In the vertebrate retina, transducin, a G-protein, binds and activates phosphodiesterase 6 (PDE6), the G-protein effector; these events result in cGMP dissociation, which in turn reduces the cation influx through cGMP-gated channels [13]. However, in *C. elegans*, there is no report of expression of the transducin or PDE6, and our BLAST searches did not recover *C. elegans* homologs of transducin or PDE6. Moreover, a recently published article [14] shows that mutants of each of five PDE genes (*pde1*–*pde5*) do not produce abnormal chemotaxis toward benzaldehyde, an attractant sensed by AWC neurons. The remaining PDE, PDE6, is described by www.wormbase.org as an isoform of mammalian cAMP-specific PDE8A. Therefore, PDE in *C. elegans* apparently differs from PDE6 in the vertebrate retina, and we proposed that in *C. elegans* guanylate cyclase (GCY), an enzyme that synthesized cGMP from GTP, was the Gα effector. We posited that, upon odor stimulation, GCY was suppressed by Gα and, upon cessation of the stimulus, the suppression was removed and cGMP synthesis was restored.

#### Synthesis and decomposition of second messenger

PDE converts cGMP to GMP. There are 11 PDE paralogs, PDE1 to PDE11, in vertebrates [15], and there are six PDE paralogs PDE-1 to PDE-6 in *C. elegans*. We confirmed that PDE-1 and PDE-3 show high homology with vertebrate PDE1 and PDE3, respectively, which catalyze cGMP decomposition. PDE1 is expressed in brain tissue, whereas PDE3 is expressed in many tissues, but not in brain [15]. Therefore, we incorporated only PDE-1 into the model. Decomposition of cGMP is modulated when Ca^2+^ ions or calmodulin bind PDE1.

#### CNG channels and voltage-gated calcium channels

CNG channels are necessary for signal transduction in AWCs. When the channel subunits TAX-2 and TAX-4 are expressed in cultured cells, both monomeric TAX-4 channels, which allow only sodium ions to pass, and heteromeric TAX-2/TAX-4 channels, which allow cations (including calcium ions) to pass, form [16]. CNG channels are directly activated by cGMP. The influx of calcium ions elevates the membrane potential; as a result, the Ca^2+^ influx activates voltage-dependent calcium channels (VDCC). Electrophysiological data are available for AWCs, unlike most *C. elegans* neurons [1]. In our model, the values for two parameters, VDCC channels and membrane currents, were set based on the current-voltage relationship evident from the physiological data. The change in membrane potential with time was calculated by using the current-voltage relationship of AWCs [1] as described in the Method section (see also Fig. S1 and the equations in ‘Membrane currents and membrane potential’ in Text S1).

#### Feedback on GCY

In order to realize a transient increase in [Ca^2+^] after removal of odor stimulus, a mechanism for transient increase in GCY activity was included in the model. The level of GCY activity should be positively correlated with length of odor stimulation. A guanylate cyclase activating protein (GCAP) was included in the feedback loop incorporated into our model. *C. elegans* NCS-1 has high homology to vertebrate GCAP; GCAP activate GCY at low calcium concentrations in vertebrate retina [17]. NCS-1 localizes in many neurons, including the AWCs. NCS-2 and NCS-3 also have high homology with GCAP, but the localization of NCS-2 and NCS-3 in neurons is not well characterized. We assume two GCAPs (GCAPa and GCAPb) support the different time courses of changes in [cGMP] that are predominantly associated with the increasing and decreasing phases, respectively, of [Ca^2+^]. GCAP potentiates GCY activity, but the effects of GCAP are suppressed when calcium ions bind GCAP; therefore, Ca^2+^-dependent negative feedback loops are formed. We posited that Gα-GCY-GCAP complexes accumulated during odor stimulation. We also hypothesized that, when the stimulus was removed, Gα dissociated from the complex, and GCY-GCAP produced cGMP; if these suppositions are true, the peak response would be dependent on the stimulus period.

#### Ca^2+^ buffer and Ca^2+^ extrusion

We theorized that calmodulin (CaM) acted as a Ca^2+^ buffer. CaM trapped free calcium ions and reduced [Ca^2+^]. Excess calcium ions were transported outside the neuron via an extrusion apparatus, possibly a sodium-calcium exchanger (CaX). We posited that CaX was regulated by a calmodulin-Ca^2+^ complex.

### Response to stimulus

**Figure 2:**
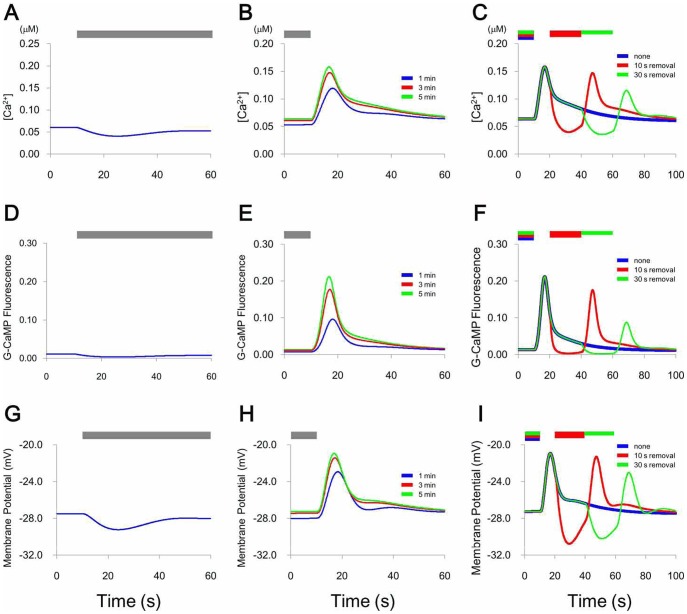
Change in [Ca^2+^]. The bars indicate periods of stimulation. A. Addition of stimulus. B. Removal of stimulus. Duration of stimulus was 1, 3, or 5 min. C. After removal of the first 5-min stimulus, a second 20-s stimulus was applied 10 or 30 s later. D, E, F. G-CaMP fluorescence associated with A, B, C, respectively. G, H, I. Membrane potential associated with A, B, C, respectively.

As shown in Fig. 1, the model included two GCAPs with different parameters and one PDE. Three requirements were imposed on the model according to Chalasani et al. [6]. 1) [Ca^2+^] must decrease following odor stimulation and increase within 1 s and reach its peak within 10 s after the odor stimulus is removed; then, [Ca^2+^] gradually returns to baseline at approximately 40 s. 2) The peak [Ca^2+^] must increase as the duration of odor stimulus increases between 1–5 min. 3) When the second stimulus is added in the period of high [Ca^2+^] after removal of the first stimulus, the [Ca^2+^] must decrease during the second stimulus. The increase in [Ca^2*^] after stimulus removal may trigger backing or turning behavior when the worm moves in the down-gradient direction of a concentration gradient of attractive odor. G-CaMP-based estimates of [Ca^2+^] depend on changes in fluorescence intensity; they do not measure [Ca^2+^] directly. Quantitative relationships between G-CaMP fluorescence and [Ca^2+^] have been assessed using a cell-free system [5] and rat pyramidal neurons [18]. However, the relationships between [Ca^2+^] and fluorescence vary widely and are dependent on experimental conditions. We adopted the EC_50_ value of G-CaMP in the cell-free system [5] for a parameter search using a genetic algorithm (see Methods). We obtained eight candidates in which the traces satisfy the three requirements imposed on the model, and analyzed further the best candidate. The time courses of simulated [Ca^2+^], G-CaMP fluorescence changes, and membrane potential changes (see Methods) upon application of odor for different periods of time are shown in Fig. 2 and Fig. S4; in some cases, a second odor stimulus was applied.

**Figure 3:**
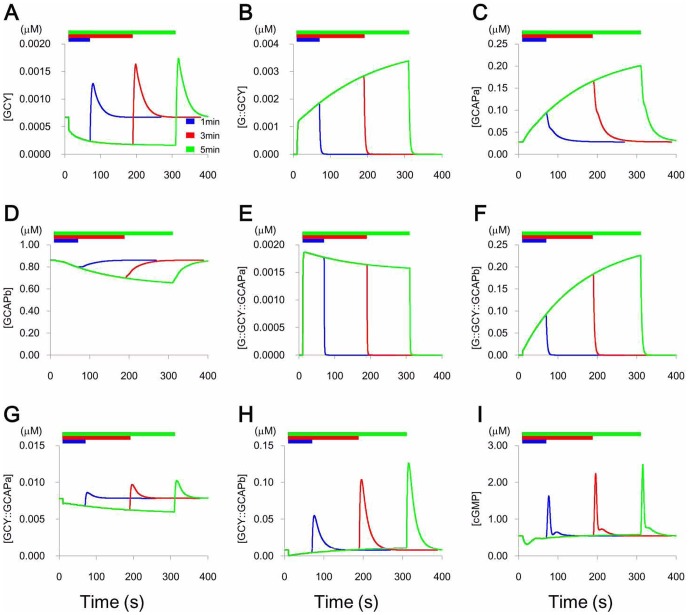
Changes in the nine components. Changes in the components, indicated in each panel, from 10 s before the application of the stimulus for 1 (blue), 3 (red) and 5 min (green).

Figure 3 shows changes in concentrations of nine intracellular components, including individual molecules and multimeric complexes, from 10 s before the application to 50 s after the removal of the stimulus. We posited that Gα bound to and inhibited GCY when a stimulus was given and that [G::GCY] increased as a result of this binding (Fig. 3A, 3B). [GCY] increased and [G::GCY] decreased following the removal of the stimulus. Calcium-free GCAPi (i = a, b) were the active forms. The active forms of the GCAPi formed under lower calcium conditions during odor stimulus (Fig. 3C, 3D). Then four complexes, GCY::GCAPi and G::GCY::GCAPi formed. G::GCY::GCAPb was accumulated during sustained (5 min) odor stimuli than during stimuli that lasted only 1 min (Fig. 3F). It was necessary that the accumulation of the inactive form of the complex, [G::GCY::GCAPb], was higher with a 5-min stimulus than with a 1-min stimulus (Fig. 3F, Table S1), to realize the peak [Ca^2+^] increased with longer exposure to the odor (Figs. 2B and S4). In contrast, accumulation of complexes that contained GCAPa was lower with a 5-min stimulus than with a 1-min stimulus. (Fig. 3E).

After removal of the stimulus, G::GCY::GCAPi rapidly dissociated to Gα and the active form GCY::GCAPi to produce cGMP (Fig. 3E–3I). GCY::GCAPb produced more cGMP than GCY::GCAPa (Table S1). [GCY::GCAPb] was always higher than [GCY::GCAPa] (Fig. 3G, 3H). Accordingly, the response after the removal of stimulus, the contribution of GCY::GCAPb::GTP to the increases in [cGMP] was larger than that of GCY::GCAPa::GTP. GCY::GCAPi::GTP and GCY::GTP produced cGMP, but the trimeric complexes produced more cGMP than the dimeric complexes (Table S1). [GCY] gradually returned to its steady-state value after removal of the stimulus (Fig. 3A). Changes in concentrations of intracellular components containing or interacting with PDE or CaM (the calcium buffer) are shown in Figures S2 and S3.

**Figure 4:**
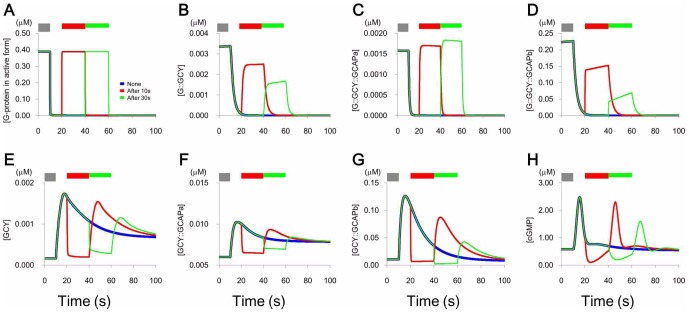
Changes in the components by addition of the second stimulus. Changes in intracellular pathway components, indicated in each panel, by addition of the second stimulus after 10 s (red) and 30 s (green) after removal of the first stimulus for 5 min.

With regard to the mechanism of [Ca^2+^] change induced by a second stimulus (Figs. 2C and S4), the receptor and Gα were activated again (Fig. 4A), and GCY complexes did convert to the inactivated forms (Fig. 4B–4D). Concentrations of GCY complexes that had enzyme activity decreased rapidly (Fig. 4E–4G), and these decreases resulted in decreases in cGMP synthesis (Fig. 4H). Thus, rates of complex dissociation overcame rates of cGMP synthesis. Consequently, [cGMP] was low during secondary stimulations, and removal of stimulus produced transient and small increases in [Ca^2+^] (Fig. 2C).

### Sensitivity to noise input

**Figure 5:**
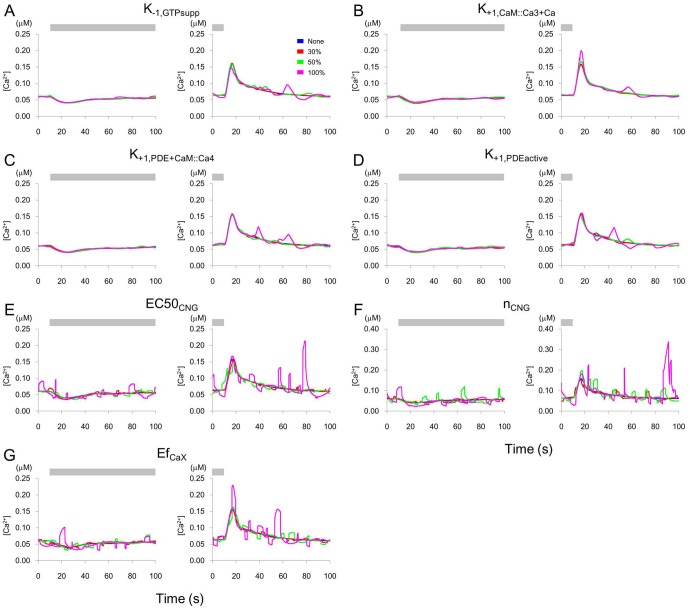
Changes in [Ca^2+^] by fluctuation of the equation parameters. Changes in [Ca^2+^] when the equation parameters (indicated in each panel) include a random pulse train as described in Methods. The fluctuation of EC50_CNG_, n_CNG_ and Ef_CaX_ produced large changes in [Ca^2+^]. Odor stimulus was given for 5 min and was removed at 10 s (right panel). The bar indicates the stimulus. Blue: no addition of fluctuation. Red, green, and magenta lines represent 30, 50, 100% magnitudes of the maximum pulse height of the noise, respectively.

Reportedly, in the decreasing phase of the response after removal of stimulus, [Ca^2+^] fluctuates [6]. These spontaneous [Ca^2+^] fluctuations in AWCs are suppressed by insulin signals, which are feedback signals from a pair of AIA interneurons [11]. Moreover, stochastic transient rises in [Ca^2+^] in response to temperature increases have been observed in AWCs [19]. However, in our model, if the external fluctuation was not added, [Ca^2+^] did not fluctuate as it decreased following odor stimulation. To examine which part of the model was sensitive to the external noise, we added a random pulse train with random widths, intervals and magnitudes to each parameter value (see Methods). If the maximal deviation of [Ca^2+^] from that without the noise was less than 5% of the resting [Ca^2+^] without odor stimulation, the parameter was regarded as insensitive to the noise. The remaining, noise-sensitive parameters were those relating to the CaM-Ca complex, GCY::GCAPb, GTP supply, PDE, CNG channel, and calcium extrusion. The reported noise activity [11] shows that [Ca^2+^] increases transiently with larger magnitudes than those of the transient [Ca^2+^] decreases. Parameters which satisfied this condition were restricted to K_−1,GTPsupp_, K_+1,CaM+Ca_, K_+1,CaM::Ca+Ca_, K_+1,CaM::Ca2+Ca_, K_+1,CaM::Ca3+Ca_; K_+1,PDE+CaM::Ca4_; K_+1,PDEactive_, EC50_CNG_, n_CNG_ and Ef_CaX_. The responses to addition of noise to these parameters are shown in Fig. 5. Other noise sensitive parameters showed that [Ca^2+^] increased with less than or similar maximal magnitude compared with that of [Ca^2+^] decrease.

**Figure 6:**
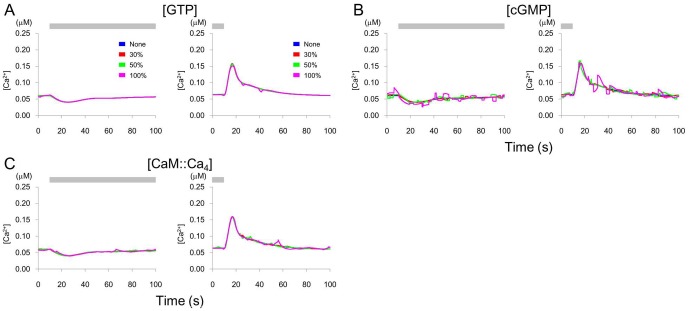
Changes in [Ca^2+^] by fluctuation of the concentrations of substances. Change in [Ca^2+^] when the concentrations of substances are determined using a random pulse train. Addition and removal of odor stimulus, and the colors of line are same as those in Fig. 5. The component is indicated in each panel. A. GTP, B. cGMP, C. CaM::Ca_4_ where Ca_4_ indicates four calcium ions.

Next, the parameters were fixed and a pulse train noise was added to concentrations of individual components of the substances (GTP, cGMP, and CaM::Ca_4_) concerning the above-mentioned sensitive parameters. CaM::Ca_4_ and cGMP showed transient [Ca^2+^] increases in the presence of noise, while GTP was insensitive to noise (Fig. 6).

**Figure 7:**
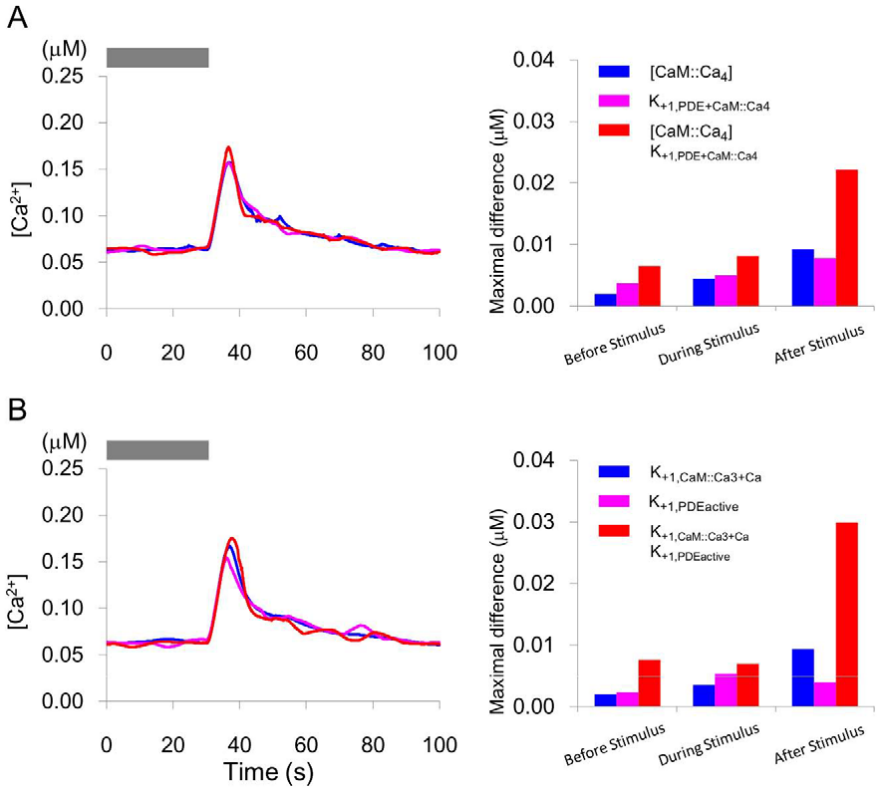
Synergistic effects of pairs of noise inputs. When pulse trains were added alongside different parameters or molecular concentrations, a synergistic increase in [Ca^2+^] fluctuations was observed. Noises were applied simultaneously to A) CaM::Ca_4_ and K_+1,PDE+CaM::Ca4_ and B) K_+1,CaM::Ca3+Ca_ and K_+1,PDEactive_. Bar indicates the maximal difference between [Ca^2+^] trace without noise and that with noise.

Independent noise trains were added for every two noise sensitive factors that are described above to examine synergistic effects on [Ca^2+^]. When the noises were applied simultaneously to CaM::Ca_4_ and K_+1,PDE+CaM::Ca4_, or K_+1,CaM::Ca3+Ca_ and K_+1,PDEactive_, fluctuations in [Ca^2+^] increased (Fig. 7).

## Discussion

We considered that responses to odors in AWCs start when the odor molecule binds to a receptor that then activates G-proteins. Moreover, cGMP may be the second messenger that mediates olfaction because cGMP-synthesis enzymes are necessary for olfaction and because CNG channels are very sensitive to cGMP and less sensitive to cAMP. However, the mechanism of cGMP synthesis in AWCs is unclear. In contrast, olfactory neurons of vertebrates show excitation by odor stimulation, and the second messenger cAMP elevates [Ca^2+^] via CNG-channels [7], [8]. [Ca^2+^] increases in AWC neurons occur following the removal of odor stimulation. This response is similar to the response of photoreceptor cells in the vertebrate retina, where light-stimulated photoreceptors activate G-transducin, a G-protein that activates PDE6, and these events result in a decrease in cGMP. However, our BLAST searches did not recover a G-transducin homolog in *C. elegans*, and there is no published evidence that any of the six PDEs in *C. elegans* are specific to olfaction. Therefore, we did not base the pathway used in our model on the scheme of the G-transducin-PDE6 pathway in vertebrate photoreceptors.

Genes *pde-1* through *pde-6* are listed in Wormbase, the *C. elegans* database. PDE-1, PDE-2, and PDE-3 are highly homologous to mammalian cGMP-hydrolysable PDE1, PDE2, and PDE3, respectively. The activity of PDE1 is regulated by calcium ions and calmodulin [15]. PDE-3 is mainly expressed in non-neuronal tissues [15], and so is not included in our model. Moreover, PDE-4 and PDE-6 are highly homologous to cAMP-specific mammalian PDE; therefore, these PDEs were not used in our model. PDE-5 is highly homologous to human PDE10A. PDE10A acts on both cAMP and cGMP but dissociates only at very high cGMP concentrations [20]. However, there is no published evidence that in *C. elegans*, odor stimulation or any odor-dependent behavioral output is associated with cAMP-dependent signal transduction or a cAMP cascade; therefore, we conclude that PDE-5 does not function in AWC responses to odor. PDE-1 and PDE-2 act on both cAMP and cGMP [15]; thus, they were both considered candidates for the PDE in the present model. However, PDE-1, closely related to Ca^2+^/calmodulin-dependent phosphodiesterases (Wormbase), was sufficient to reproduce the experimental data. To date, there is no report that Gα suppresses GCY, and GCY may be suppressed either directly or indirectly by Gα; however, for simplicity we posited direct suppression. We examined also whether a model with one GCAP and two PDEs could produce the [Ca^2+^] changes shown in Fig. 2. However, such a model did not realize the rapid rise and slow decay, and the decrease of [Ca^2+^] by the second odor stimulus. Therefore, two GCAP and one PDE were used in the model.

A negative feedback pathway was essential for transient increases in [Ca^2+^] that had magnitudes that were dependent on the duration of the stimulus. We chose GCAP as the feedback factor for cGMP synthesis. However, GCAP could be replaced by another protein while maintaining the basic feedback scheme; for example, the type II calmodulin-dependent protein kinase UNC-43 modulates the function of other proteins in a [Ca^2+^]-dependent manner, although the feedback loop has to be modified. Moreover, accumulation of the Gα-GCY-GCAP complex during odor stimulation was included in the model. Experimental studies are necessary to verify the model.

Spontaneous [Ca^2+^] activity in AWCs is observed experimentally, and is suppressed by a neuropeptide-mediated feedback from AIA interneurons [11]. In our model, [Ca^2+^] fluctuations were only observed when external input was applied. It is possible that the fluctuations observed in imaging experiments may result from synaptic inputs or from external disturbances of the intracellular signaling pathway. The parameters, which satisfied the condition that noise activity shows transient large [Ca^2+^] increases and only small decreases [11], were restricted to EC50_CNG_, n_CNG_; K_−1,GTPsupp_; K_+1,CaM+Ca_, K_+1,CaM::Ca+Ca_, K_+1,CaM::Ca2+Ca_, K_+1,CaM::Ca3+Ca_; K_+1,PDE+CaM::Ca4_; K_+1,PDEactive_ and Ef_CaX_. These parameters, and therefore the decrease in GTP supply, the increase in concentration of active PDE to bind cGMP, or the calcium extrusion mechanism, might concern the spontaneous activity in AWC neurons. For example, EC50_CNG_ is affected by calmodulin [21] of which fluctuation of binding may induce spontaneous [Ca^2+^] activity. EC50_CNG_ and n_CNG_ were hyper-sensitive to noise (Fig. 5) and might be predicted to require tight regulation. Also, some pairs of noise inputs had synergistic effects on certain parameters or molecular concentrations. Although not included in our model, the extrusion of Ca^2+^ ions from the intracellular calcium store, or synaptic or peptidergic inputs from outside the AWC, might be a source of the spontaneous activity. Chalasani et al. (2010) reported that AWC releases both glutamate and the neuropeptide NLP-1. These transmitters are sensed by AIA interneurons that, when odor is present, release the insulin-like neuropeptide INS-1, which suppresses AWC calcium transients. Although the INS-1 receptors in AWC and their downstream molecules are not yet identified, the whole model of [Ca^2+^] activity should be constructed in the future based on experimental studies.

We have presented a hypothetical model that can explain the changes in [Ca^2+^] that occur in AWC neurons upon odor stimulation. We have also used this model to analyze concentration changes in molecules and to identify possible causes of [Ca^2+^] fluctuation.

## Methods

### Reaction formula

Activation of second messenger proteins and their enzyme reactions were modeled using differential equations in which the mass-action law (i.e., the condition that binding rates between different chemical species are proportional to their concentrations) is supposed for all processes, if not stated explicitly. The differential equations are presented in Text S1. Changes in calcium current via CNG channels [16] and VDCC calcium ion channels [22] were faster than the change in [Ca^2+^] in AWCs following odor stimulation [6]. Moreover, the sodium and calcium exchanger in olfactory sensory neurons rapidly extrudes calcium ions [23]. Thus, for simplicity, changes in the voltage and ion concentrations caused by Ca^2+^ influx and efflux were described by Hill equations or modified Hill equations formulated by the authors. Relationship between G-CaMP fluorescence and [Ca^2+^] (Text S1) was based on [5]. Membrane potential was also calculated using the current-voltage relationship of AWCs [1] (see Fig. S1) and the equations shown in ‘Membrane currents and membrane potential’ in Text S1. We used COPASI software (http://www.copasi.org) to perform these simulations (COPASI-file S1).

### Determination of parameters

The resting membrane potential in AWCs is approximately −28 mV [1]. We used these values in our model. Parameters for CNG channels, current-voltage relationship of VDCC, and current-voltage relationship of the whole current (Fig. S1) were set based on published electrophysiological data [16], [22] and [1], respectively. Other parameter values in the equations were determined using the genetic algorithm software implemented in COPASI, to reflect calcium imaging data for G-CaMP-expressing AWC neurons [6], under the condition that the membrane potential ranges from −80–+20 mV, and concentrations of GTP and cGMP are 0.001–10 µM. The range for cGMP was based on its concentration in retina [24]. Other parameters ranged as shown in Table S1. Table S1 includes the values used for figures in the result section.

### Addition of external noise

The pulse train noise with the following features was made. The pulse height was A(1+R). A was fixed to the parameter value when the noise was added to the parameter value, whereas A was variable (the concentration value in the case without noise) whenever the noise was added to the concentration of any substance. R was the uniform noise with the range [−1, 1]. Pulse width had a uniform random value between 1 and 5 s. The interval between the end of a pulse and the start of the succeeding pulse was a uniform random value between 0.1 and 10 s.

## Supporting Information

COPASI-file S1 COPASY-file including the information that is necessary to perform simulation.
(CPS)

Figure S1 Current-voltage relationship for voltage-dependent currents without odor stimulation in AWC neurons. Deduced from electrophysiological data published by Nickell et al. (2002).
(PDF)

Figure S2 Changes in concentrations of intracellular pathway components. These changes are related to PDE and catalytic reactions caused by the addition or removal of odor stimulation. A. PDE, B. PDE::cGMP, C. PDE_active_ ( = PDE::CaM::Ca_4_, where Ca_4_ indicates four calcium ions), D. PDE_active_::cGMP.
(PDF)

Figure S3 Changes in concentrations of intracellular pathway components. These were related to a combination of calcium buffer and calcium ions caused by addition or removal of odor stimulation. A. Free calmodulin (CaM)-like calcium buffer, B. CaM::Ca_1_, C. CaM::Ca_2_, D. CaM::Ca_3_, E. CaM::Ca_4_. CaM::Ca_k_ (k = 1–4) calmodulin binding with k calcium ions.
(PDF)

Figure S4 Changes in [Ca^2+^], G-CaMP fluorescence and membrane potential. The bars indicate periods of stimulation. A, B, C. Responses to 1-min stimulus. D, E, F. After removal of the first 1-min stimulus, a second 20-s stimulus was applied 10 s later. G, H, I. After removal of the first 1-min stimulus, a second was applied 30 s later. J, K, L. Responses to 5-min stimulus. M, N, O. After removal of the first 5-min stimulus, a second 20-s stimulus was applied 10 s later. P, Q, R. After removal of the first 5-min stimulus, a second was applied 30 s later.
(PDF)

Table S1 Values of parameters in equations. This table includes the values used for figures in the result section, the lower and upper limits used in the genetic algorithm, and the reference numbers in References in the text.
(PDF)

Text S1 List of equations.
(PDF)
