# Supplementary figures and images for: A Model of the Intracellular Response of an Olfactory Neuron in Caenorhabditis elegans to Odor Stimulation
Source: PLoS One. 2012 Aug 23;7(8):e42907. doi: 10.1371/journal.pone.0042907 (PMC3426523; doi:10.1371/journal.pone.0042907)

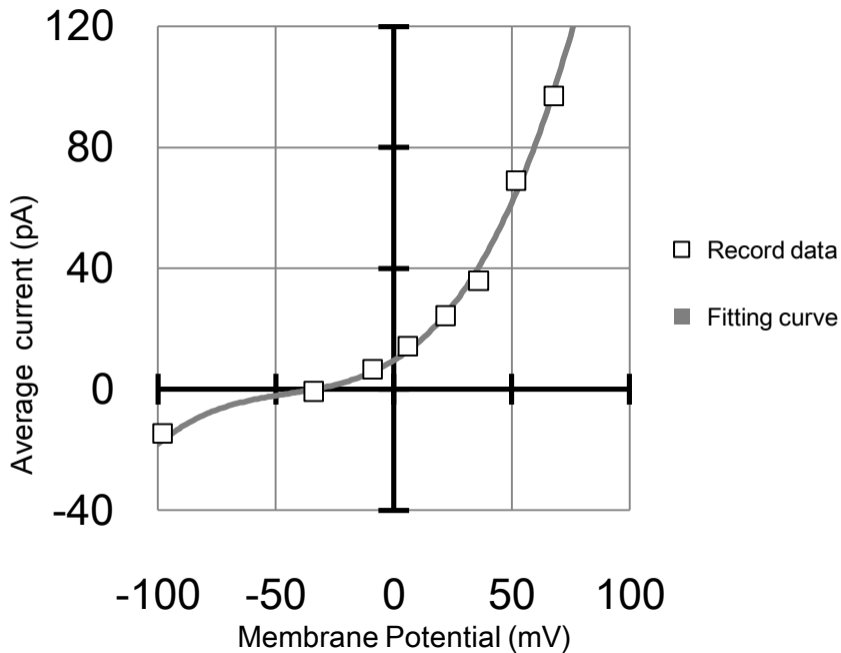

Supplement: Figure S1 — Current-voltage relationship for voltage-dependent currents without odor stimulation in AWC neurons. Deduced from electrophysiological data published by Nickell et al. (2002). (PDF) [file pone.0042907.s002.pdf]

**A**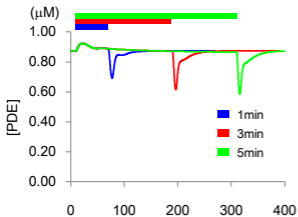**B**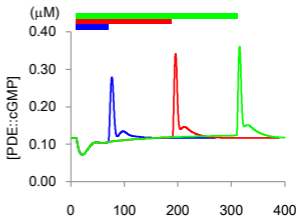**C**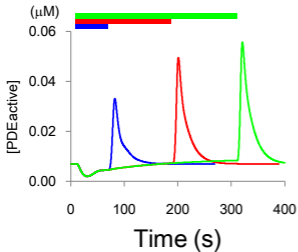**D**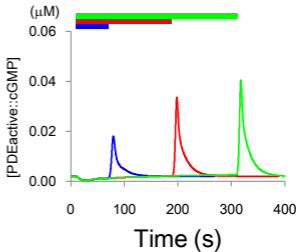

Supplement: Figure S2 — Changes in concentrations of intracellular pathway components. These changes are related to PDE and catalytic reactions caused by the addition or removal of odor stimulation. A. PDE, B. PDE::cGMP, C. PDEactive ( = PDE::CaM::Ca4, where Ca4 indicates four calcium ions), D. PDEactive::cGMP. (PDF) [file pone.0042907.s003.pdf]

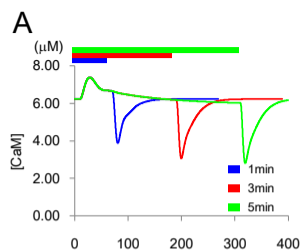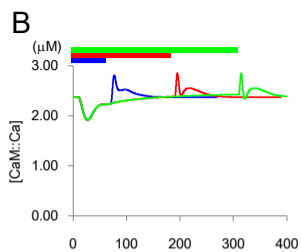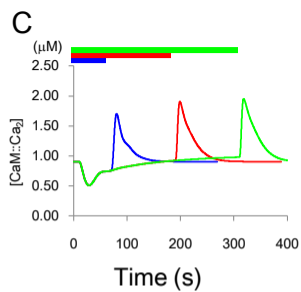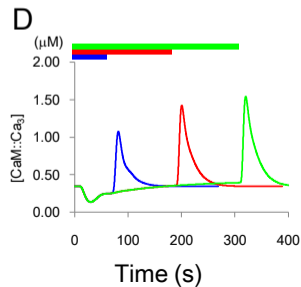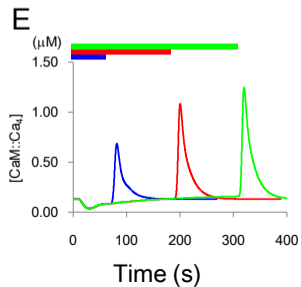

Supplement: Figure S3 — Changes in concentrations of intracellular pathway components. These were related to a combination of calcium buffer and calcium ions caused by addition or removal of odor stimulation. A. Free calmodulin (CaM)-like calcium buffer, B. CaM::Ca1, C. CaM::Ca2, D. CaM::Ca3, E. CaM::Ca4. CaM::Cak (k = 1–4) calmodulin binding with k calcium ions. (PDF) [file pone.0042907.s004.pdf]

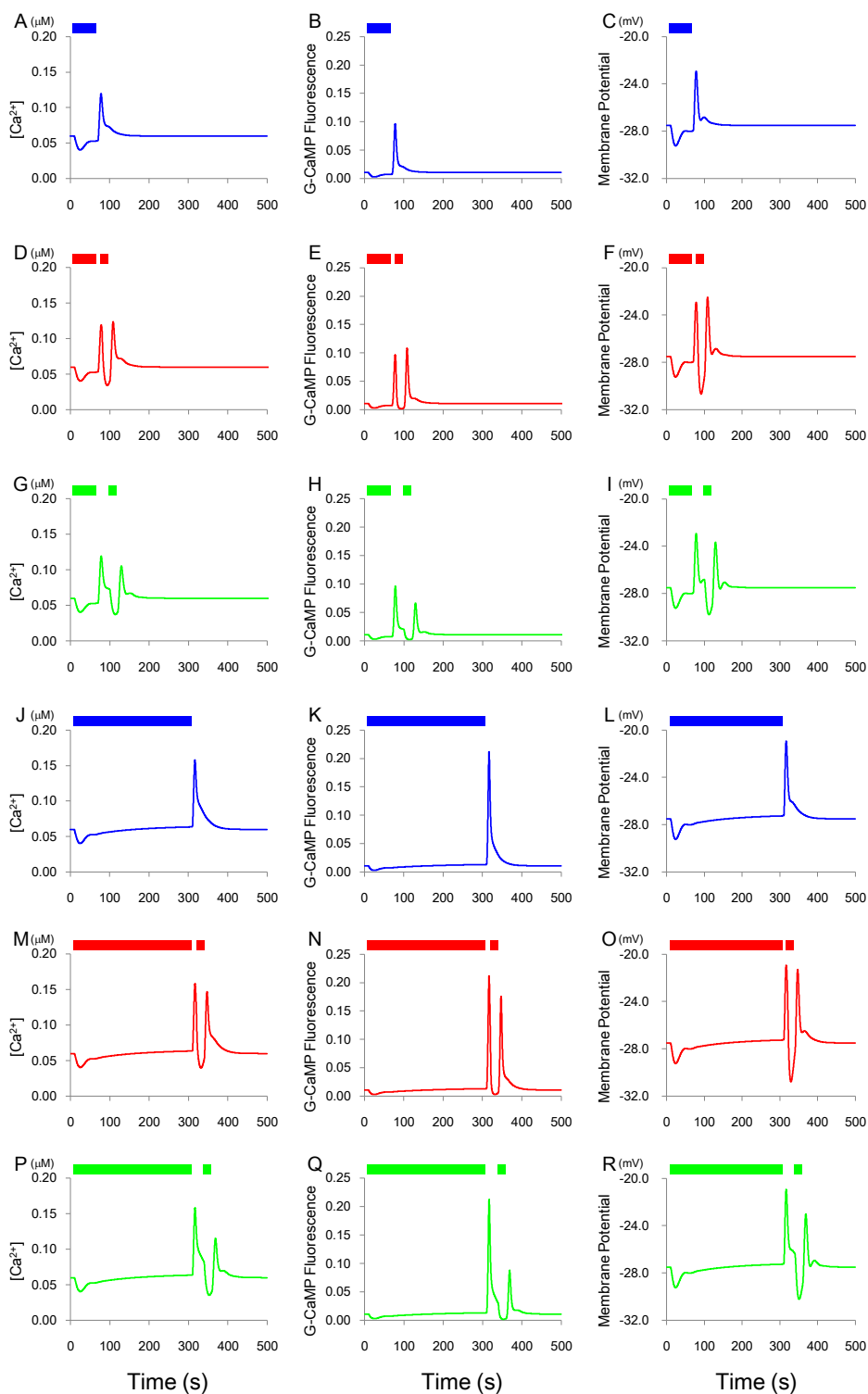

Supplement: Figure S4 — Changes in [Ca2+], G-CaMP fluorescence and membrane potential. The bars indicate periods of stimulation. A, B, C. Responses to 1-min stimulus. D, E, F. After removal of the first 1-min stimulus, a second 20-s stimulus was applied 10 s later. G, H, I. After removal of the first 1-min stimulus, a second was applied 30 s later. J, K, L. Responses to 5-min stimulus. M, N, O. After removal of the first 5-min stimulus, a second 20-s stimulus was applied 10 s later. P, Q, R. After removal of the first 5-min stimulus, a second was applied 30 s later. (PDF) [file pone.0042907.s005.pdf]
