# Supplementary material for: A Model of the Intracellular Response of an Olfactory Neuron in Caenorhabditis elegans to Odor Stimulation
Source: PLoS One. 2012 Aug 23;7(8):e42907. doi: 10.1371/journal.pone.0042907 (PMC3426523; doi:10.1371/journal.pone.0042907)
Supplement: Table S1 — Values of parameters in equations. This table includes the values used for figures in the result section, the lower and upper limits used in the genetic algorithm, and the reference numbers in References in the text. (PDF) [file pone.0042907.s006.pdf]

Parameters

	Parameter	Symbol	Value *)	Unit
Receptor	Activation rate for receptor	$K_{+1,R}$	0.50 (0.001-1000)	1/(µM*s)
	Inactivation rate for receptor	$K_{-1,R}$	45.40 (0.001-1000)	s^{-1}
G-Protein	Activation rate for G protein	$K_{+1,G}$	10.50 (0.001-1000)	1/(µM*s)
	Inactivation rate for G protein	$K_{-1,G}$	15.20 (0.001-1000)	s^{-1}
GCAPa	Binding rate for GCAPa + Ca	$K_{+1,GCAPa+Ca}$	6.48 (0.001-1000)	1/(µM*s)
	Dissociation rate for GCAPa::Ca into GCAPa and Ca	$K_{-1,GCAPa+Ca}$	1.70 (0.001-1000)	s^{-1}
GCAPb	Binding rate for GCAPb+Ca	$K_{+1,GCAPb+Ca}$	0.098 (0.001-1000)	1/(µM*s)
	Dissociation rate for Ca and GCAPb from GCAPb::Ca	$K_{-1,GCAPb+Ca}$	0.0428 (0.001-1000)	s^{-1}
Buffer	Binding rate for CaM + Ca	$K_{+1,CaM+Ca}$	4.06 (0.001-1000)	1/(µM*s)
	Dissociation rate for CaM::Ca1 into CaM and Ca	$K_{-1,CaM+Ca}$	0.638 (0.001-1000)	s^{-1}
	Binding rate for CaM::Ca_1 + Ca	$K_{+1,CaM::Ca1+Ca}$	4.06 (0.001-1000)	1/(µM*s)
	Dissociation rate for CaM::Ca_2 into CaM::Ca_1 and Ca	$K_{-1,CaM::Ca1+Ca}$	0.638 (0.001-1000)	s^{-1}
	Binding rate for CaM::Ca_2 + Ca	$K_{+1,CaM::Ca2+Ca}$	4.06 (0.001-1000)	1/(µM*s)
	Dissociation rate for CaM::Ca_3 into CaM::Ca_2 and Ca	$K_{-1,CaM::Ca2+Ca}$	0.638 (0.001-1000)	s^{-1}
	Binding rate for CaM::Ca_3 + Ca	$K_{+1,CaM::Ca3+Ca}$	4.06 (0.001-1000)	1/(µM*s)
	Dissociation rate for CaM::Ca_4 into CaM::Ca_3 and Ca	$K_{-1,CaM::Ca3+Ca}$	0.638 (0.001-1000)	s^{-1}

	Parameter	Symbol	Value	Unit
GCY	Binding rate for GCY + Gα	$K_{+1,GCY+G}$	0.158 (0.001-1000)	1/(μM*s)
	Dissociation for G::GCY into Gα and GCY	$K_{-1,GCY+G}$	0.012 (0.001-1000)	s^{-1}
	cGMP production by GCY GCY+GTP binding	$K_{+1,GCY}$	0.139 (0.001-1000)	1/(μM*s)
	cGMP production by GCY GCY::GTP dissociation	$K_{-1,GCY}$	0.021 (0.001-1000)	s^{-1}
	cGMP production by GCY cGMP production	$K_{+2,GCY}$	0.010 (0.001-1000)	s^{-1}
GCY::GCAPa	Binding rate for GCY+GCAPa	$K_{+1,GCY+GCAPa}$	50.800 (0.001-1000)	1/(μM*s)
	Dissociation for GCY::GCAPa into GCY and GCAPa	$K_{-1,GCY+GCAPa}$	0.120 (0.001-1000)	s^{-1}
	Binding rate for GCY::GCAPa + Gα	$K_{+1,GCY::GCAPa+G}$	1.010 (0.001-1000)	1/(μM*s)
	Dissociation for G::GCY::GCAPa into Gα and GCY::GCAPa	$K_{-1,GCY::GCAPa+G}$	0.730 (0.001-1000)	s^{-1}
	Binding rate for G::GCY+GCAPa	$K_{+1,G::GCY+GCAPa}$	1.010 (0.001-1000)	1/(μM*s)
	Dissociation for G::GCY::GCAPa into G::GCY and GCAPa	$K_{-1,G::GCY+GCAPa}$	0.730 (0.001-1000)	s^{-1}
	cGMP production by GCY::GCAPa GCY::GCAPa+GTP binding	$K_{+1,GCY::GCAPa}$	0.019 (0.001-1000)	1/(μM*s)
	cGMP production by GCY::GCAPa GCY::GCAPa::GTP dissociation	$K_{-1,GCY::GCAPa}$	0.011 (0.001-1000)	s^{-1}
	cGMP production by GCY::GCAPa cGMP production	$K_{+2,GCY::GCAPa}$	0.012 (0.001-1000)	s^{-1}
GCY::GCAPb	Binding rate for GCY+GCAPb	$K_{+1,GCY+GCAPb}$	1.35 (0.001-1000)	1/(μM*s)
	Dissociation for GCY::GCAPb into GCY and GCAPb	$K_{-1,GCY+GCAPb}$	0.100 (0.001-1000)	s^{-1}
	Binding rate for GCY::GCAPb + Gα	$K_{+1,GCY::GCAPb+G}$	1.01	1/(μM*s)

			(0.001-1000)	
	Dissociation for G::GCY::GCAPb into Gα and GCY::GCAPb	$K_{-1,GCY::GCAPb+G}$	0.730 (0.001-1000)	s^{-1}
	Binding rate for G::GCY+GCAPb	$K_{+1,G::GCY+GCAPb}$	1.55 (0.001-1000)	1/(μM*s)
	Dissociation for G::GCY::GCAPb into G::GCY and GCAPb	$K_{-1,G::GCY+GCAPb}$	0.011 (0.001-1000)	s^{-1}
	cGMP production by GCY::GCAPb GCY::GCAPb+GTP binding	$K_{+1,GCY::GCAPb}$	1.10 (0.001-1000)	1/(μM*s)
	cGMP production by GCY::GCAPb GCY::GCAPb::GTP dissociation	$K_{-1,GCY::GCAPb}$	0.0132 (0.001-1000)	s^{-1}
	cGMP production by GCY::GCAPb cGMP production	$K_{+2,GCY::GCAPb}$	25.800 (0.001-1000)	s^{-1}

	Parameter	Symbol	Value	
PDE	cGMP decomposition by PDE PDE+cGMP binding	$K_{+1,PDE}$	1.73 (0.001-1000)	1/(μM*s)
	cGMP decomposition by PDE PDE::cGMP dissociation	$K_{-1,PDE}$	6.99 (0.001-1000)	s^{-1}
	cGMP decomposition by PDE cGMP decomposition	$K_{+2,PDE}$	0.0136 (0.001-1000)	s^{-1}
PDEactive	Binding rate for PDE + CaM::Ca_4	$K_{+1,PDE+CaM::Ca4}$	30.480 (0.001-1000)	1/(μM *s)
	Inactivation rate for PDEactive into PDE and CaM::Ca_4	$K_{-1,PDE+CaM::Ca4}$	500.610 (0.001-1000)	s^{-1}
	cGMP decomposition by PDEactive PDEactive +cGMP binding	$K_{+1,PDEactive}$	19.7 (0.001-1000)	1/(μM *s)
	cGMP decomposition by PDEactive PDEactive::cGMP dissociation	$K_{-1,PDEactive}$	0.014 (0.001-1000)	s^{-1}
	cGMP decomposition by PDEactive cGMP decomposition	$K_{+2,PDEactive}$	36.4 (0.001-1000)	s^{-1}

	Parameter	Symbol	Value	Unit
GTP supply	GTP supply	$K_{+1,GTPsupp}$	37.3 (0.01-100)	μM /s
	GTP decomposition	$K_{-1,GTPsupp}$	4.98 (0.01-100)	s^{-1}

	Parameter	Symbol	Value	Unit
CNG channel	Maximum voltage change via CNG	$I_{CNG,max}$	1.00 (0.1-10)	mV/s
	EC_{50} for CNG	$EC50_{CNG}$	8.4 [16]	μM
	Hill coefficient for CNG	n_{CNG}	0.97 [16]	-
	Reversal potential of CNG	$V_{R,CNG}$	0.08 [16]	mV
	--	Ef_{CNG}	1.00 (0.1-2.0)	μM/s
VGCC	Slope of voltage dependence of VGCC	K_{VGCC}	4.0 [22]	mV
	Reversal potential of VGCC	$V_{R,VGCC}$	30 (25-35) [22]	mV
	Half maximal voltage of VGCC	$V_{50,VGCC}$	-10 (-15-(-5)) [22]	mV
	--	Ef_{VGCC}	1.0	μM /s
VG channel	Efficiency for VG channel	Ef_{VG}	2.0	mV/s
CaX	--	Ef_{CaX}	67.5 (0.01-100)	μM /s
	Hill constant for CaX	K_{CaX}	1.90 (0.01-100)	μM
	Hill coefficient for CaX	n_{CaX}	0.1 (0.1-10)	-

*) A (B - C) means that A is the value which was used in the model; B and C are the lower and upper limits used in the genetic algorithm, respectively. [n] indicates the reference number in References in the text.

Initial Values

	Symbol	Value	Unit
Odor molecule	Odorant	1.00×10^{-4}	μM
Odorant receptor total value	R_{total}	1.00	μM
Odorant receptor active form	R_{active}	1.10×10^{-5}	μM
G-protein α subunit total value	G_{total}	1.00	μM
G-protein α subunit active form	G_{active}	7.66×10^{-6}	μM
Guanylate cyclase activating protein	GCAPa	2.27×10^{-2}	μM
	GCAPa::Ca	6.34×10^{-3}	μM
	GCAPb	0.86	μM
	GCAPb::Ca	0.13	μM
Calcium buffer	CaM	6.24	μM
	CaM::Ca	2.37	μM
	CaM::Ca2	0.90	μM
	CaM::Ca3	0.34	μM
	CaM::Ca4	0.13	μM
Guanylate cyclase	GCY	6.68×10^{-4}	μM
	G::GCY	4.82×10^{-8}	μM
	GCY::GTP	2.31×10^{-2}	μM
	GCY::GCAPa	1.07×10^{-2}	μM
	G::GCY::GCAPa	4.01×10^{-8}	μM
	GCY::GCAPa::GTP	0.96	μM
	GCY::GCAPb	7.76×10^{-3}	μM
	G::GCY::GCAPb	3.27×10^{-6}	μM
	GCY::GCAPb::GTP	2.47×10^{-3}	μM
Phosphodiestelase	PDE	0.87	μM
	PDE::cGMP	0.12	μM
	PDEactive	6.97×10^{-3}	μM
	PDEactive::cGMP	2.04×10^{-3}	μM
GTP	GTP	7.47	μM
cGMP	cGMP	0.54	μM
Membrane Potential	$V_{Membrane}$	-27.50	mV
Calcium ion	Ca	6.00×10^{-2}	μM
