# Supplementary material for: A Model of the Intracellular Response of an Olfactory Neuron in Caenorhabditis elegans to Odor Stimulation
Source: PLoS One. 2012 Aug 23;7(8):e42907. doi: 10.1371/journal.pone.0042907 (PMC3426523; doi:10.1371/journal.pone.0042907)
Supplement: Text S1 — List of equations. (PDF) [file pone.0042907.s007.pdf]

Equations

Receptor activation

$$\frac{d[R_{active}]}{dt}=K_{+1,R}[odorant]([R_{total}]-[R_{active}])-K_{-1R}[R_{active}]$$

G-protein active form

$$\frac{d[G_{active}]}{dt}=K_{+1,G}[R_{active}]([G_{total}]-[G_{active}])-K_{-1G}[G_{active}]$$

$$-K_{+1,GCY+G}[GCY][G_{active}]+K_{-1,GCY+G}[G::GCY]$$

$$-K_{+1,GCY::GCAPa+G}[GCY::GCAPa][G_{active}]+K_{-1,GCY::GCAPa+G}[G::GCY::GCAPa]$$

$$-K_{+1,GCY::GCAPb+G}[GCY::GCAPb][G_{active}]+K_{-1,GCY::GCAPb+G}[G::GCY::GCAPb]$$

GCAPa and GCAPb

$$\frac{d[GCAPa]}{dt}=-K_{+1,GCAPa+Ca}[GCAPa][Ca]+K_{-1,GCAPa+Ca}[GCAPa::Ca]$$

$$-K_{+1,GC+GCAPa}[GCY][GCAPa]+K_{-1,GCY+GCAPa}[GCY::GCAPa]$$

$$-K_{+1,G::GCY+GCAPa}[G::GCY][GCAPa]+K_{-1,G::GCY+GCAPa}[G::GCY::GCAPa]$$

$$\frac{d[GCAPa::Ca]}{dt}=K_{+1,GCAPa+Ca}[GCAPa][Ca]-K_{-1,GCAPa+Ca}[GCAPa::Ca]$$

$$\frac{d[GCAPb]}{dt}=-K_{+1,GCAPb+Ca}[GCAPb][Ca]+K_{-1,GCAPb+Ca}[GCAPb::Ca]$$

$$-K_{+1,GC+GCAPb}[GCY][GCAPb]+K_{-1,GCY+GCAPb}[GCY::GCAPb]$$

$$-K_{+1,G::GCY+GCAPb}[G::GCY][GCAPb]+K_{-1,G::GCY+GCAPb}[G::GCY+GCAPb]$$

$$\frac{d[GCAPb::Ca]}{dt}=K_{+1,GCAPb+Ca}[GCAPb][Ca]-K_{-1,GCAPb+Ca}[GCAPb::Ca]$$

Calcium buffers

$$\frac{d[CaM]}{dt} = -K_{+1,CaM+Ca1}[CaM][Ca] + K_{-1,CaM+Ca1}[CaM::Ca_1]$$

$$\frac{d[CaM::Ca_1]}{dt} = K_{+1,CaM+Ca1}[CaM][Ca] - K_{-1,CaM+Ca1}[CaM::Ca_1]$$

$$-K_{+1,CaM::Ca1+Ca}[CaM::Ca_1][Ca] + K_{-1,CaM::Ca1+Ca}[CaM::Ca_2]$$

$$\frac{d[CaM::Ca_2]}{dt} = K_{+1,CaM::Ca1+Ca}[CaM::Ca_1][Ca] - K_{-1,CaM::Ca1+Ca}[CaM::Ca_2]$$

$$-K_{+1,CaM::Ca2+Ca}[CaM::Ca_2][Ca] + K_{-1,CaM::Ca2+Ca}[CaM::Ca_3]$$

$$\frac{d[CaM::Ca_3]}{dt} = K_{+1,CaM::Ca2+Ca}[CaM::Ca_2][Ca] - K_{-1,CaM::Ca2+Ca}[CaM::Ca_3]$$

$$-K_{+1,CaM::Ca3+Ca}[CaM::Ca_3][Ca] + K_{-1,CaM::Ca3+Ca}[CaM::Ca_4]$$

$$\frac{d[CaM::Ca_4]}{dt} = K_{+1,CaM::Ca3+Ca}[CaM::Ca_3][Ca] - K_{-1,CaM::Ca3+Ca}[CaM::Ca_4]$$

$$-K_{+1,PDE+CaM::Ca4}[PDE][CaM::Ca_4] + K_{-1,PDE+CaM::Ca4}[PDE_{active}]$$

GTP

$$\frac{d[GTP]}{dt}=K_{+1,GTPsuppli}-K_{-1,GTPsuppli}[GTP]-K_{+1,GCY}[GCY][GTP]+K_{-1,GCY}[GCY::GTP]$$

$$-K_{+1,GCY}[GCY][GTP]+K_{-1,GCY}[GCY::GTP]$$

$$-K_{+1,GCY::GCAPa}[GCY::GCAPa][GTP]+K_{-1,GCY::GCAPa}[GCY::GCAPa::GTP]$$

$$-K_{+1,GCY::GCAPb}[GCY::GCAPb][GTP]+K_{-1,GCY::GCAPb}[GCY::GCAPb::GTP]$$

cGMP

$$\frac{d[cGMP]}{dt}= K_{+2.GCY}[GCY::GTP]+K_{+2,GCY::GCAPa}[GCY::GCAPa::GTP]+K_{+2,GCY::GCAPb}[GCY::GCAPb::GTP]$$

$$-K_{+1,PDE}[PDE][cGMP]+K_{-1,PDE}[PDE::cGMP]$$

$$-K_{+1,PDEactive}[PDE_{active}][cGMP]+K_{-1,PDEactive}[PDE_{active}::cGMP]$$

Calcium ion

$$\frac{d[Ca^{2+}]}{dt} = Ef_{CNG} \frac{I_{CNG,Max}[cGMP]^{n_{CNG}}}{EC50_{CNG}^{n_{CNG}} + [cGMP]^{n_{CNG}}} (V_{R,CNG} - [Vm])$$

$$+ \frac{Ef_{VGCC}(V_{R,VGCC} - [Vm])}{\left\{1 + \exp\left(\frac{V_{50,VGCC} - [Vm]}{k_{VGCC}}\right)\right\}}$$

$$- \frac{Ef_{CaX}[Ca]}{\left\{1 + \left(\frac{CaM::Ca_4}{K_{CaX}}\right)^{n_{CaX}}\right\}}$$

$$-K_{+1,GCAPa+Ca}[GCAPa][Ca] + K_{-1,GCAPa+Ca}[GCAPa::Ca]$$

$$-K_{+1,GCAPb+Ca}[GCAPb][Ca] + K_{-1,GCAPb+Ca}[GCAPb::Ca]$$

$$-K_{+1,CaM+Ca_1}[CaM][Ca] + K_{-1,CaM+Ca_1}[CaM::Ca_1]$$

$$-K_{+1,CaM::Ca1+Ca}[CaM::Ca_1][Ca] + K_{-1,CaM::Ca1+Ca}[CaM::Ca_2]$$

$$-K_{+1,CaM::Ca2+Ca}[CaM::Ca_2][Ca] + K_{-1,CaM::Ca2+Ca}[CaM::Ca_3]$$

$$-K_{+1,CaM::Ca3+Ca}[CaM::Ca_3][Ca] - K_{-1,CaM::Ca3+Ca}[CaM::Ca_4]$$

GCY

$$\frac{d[GCY]}{dt}=-K_{+1,GCY}[GCY][GTP]+K_{-1,GCY}[GCY::GTP]+K_{+2,GCY}[GCY::GTP]$$

$$-K_{+1,GCY+G}[GCY][G_{active}]+K_{-1,GCY+G}[G::GCY]$$

$$-K_{+1,GCY+GCAPa}[GCY][GCAPa]+K_{-1,GCY+GCAPa}[GCY::GCAPa]$$

$$-K_{+1,GCY+GCAPb}[GCY][GCAPb]+K_{-1,GCY+GCAPb}[GCY::GCAPb]$$

$$\frac{d[GCY::GTP]}{dt}=K_{+1,GCY}[GCY][GTP]-K_{-1,GCY}[GCY::GTP]-K_{+2,GCY}[GCY::GTP]$$

$$\frac{d[G::GCY]}{dt}=K_{+1,GCY+G}[GCY][G_{active}]-K_{-1,GCY+G}[G::GCY]$$

GCY::GCAPa

$$\frac{d[GCY::GCAPa]}{dt}=K_{+1,GCY+GCAPa}[GCY][GCAPa]-K_{-1,GCY+GCAPa}[GCY::GCAPa]$$

$$-K_{+1,GCY::GCAPa}[GCY::GCAPa][GTP]+K_{-1,GCY::GCAPa}[GCY::GCAPa::GTP]$$

$$+K_{+2,GCY::GCAPa}[GCY::GCAPa::GTP]$$

$$-K_{+1,G+GCY::GCAPa}[GCY::GCAPa][G_{active}]+K_{-1,G+GCY::GCAPa}[G::GCY::GCAPa]$$

$$\frac{d[GCY::GCAPa::GTP]}{dt}=K_{+1,GCY::GCAPa}[GCY::GCAPa][GTP]-K_{-1,GCY::GCAPa}[GCY::GCAPa::GTP]$$

$$-K_{+2,GCY::GCAPa}[GCY::GCAPa::GTP]$$

$$\frac{d[G::GCY::GCAPa]}{dt}=K_{+1,G::GCY+GCAPa}[G::GCY][GCAPa]-K_{-1,G::GCY+GCAPa}[G::GCY::GCAPa]$$

$$+K_{+1,G+GCY::GCAPa}[GCY::GCAPa][G_{active}]-K_{-1,G+GCY::GCAPa}[G::GCY::GCAPa]$$

GCY::GCAPb

$$\frac{d[GCY::GCAPb]}{dt}=K_{+1,GCY+GCAPb}[GCY][GCAPb]-K_{-1,GCY+GCAPb}[GCY::GCAPb]$$

$$-K_{+1,GCY::GCAPb}[GCY::GCAPb][GTP]+K_{-1,GCY::GCAPb}[GCY::GCAPb::GTP]$$

$$+K_{+2,GCY::GCAPb}[GCY::GCAPb::GTP]$$

$$-K_{+1,G+GCY::GCAPb}[GCY::GCAPb][G_{active}]+K_{-1,G+GCY::GCAPb}[G::GCY::GCAPb]$$

$$\frac{d[GCY::GCAPb::GTP]}{dt}=K_{+1,GCY::GCAPb}[GCY::GCAPb][GTP]-K_{-1,GCY::GCAPb}[GCY::GCAPb::GTP]$$

$$-K_{+2,GCY::GCAPb}[GCY::GCAPb::GTP]$$

$$\frac{d[G::GCY::GCAPb]}{dt}=K_{+1,G::GCY+GCAPb}[G::GCY][GCAPb]-K_{-1,G::GCY+GCAPb}[G::GCY::GCAPb]$$

$$+K_{+1,G+GCY::GCAPb}[GCY::GCAPb][G_{active}]-K_{-1,G+GCY::GCAPb}[G::GCY::GCAPb]$$

PDE

$$\frac{d[PDE]}{dt} = -K_{+1,PDE}[PDE][cGMP] + K_{-1,PDE}[PDE::cGMP] + K_{+2,PDE}[PDE::cGMP]$$

$$-K_{+1,PDE+CaM::Ca4}[PDE][CaM::Ca_4] + K_{-1,PDE+CaM::Ca4}[PDE_{active}]$$

$$\frac{d[PDE::cGMP]}{dt} = +K_{+1,PDE}[PDE][cGMP] - K_{-1,PDE}[PDE::cGMP] - K_{+2,PDE}[PDE::cGMP]$$

PDE active form

$$\frac{d[PDE_{active}]}{dt} = -K_{+1,PDEactive}[PDE_{active}][cGMP] + K_{-1,PDEactive}[PDE_{active}::cGMP] + K_{+2,PDEactive}[PDE_{active}::cGMP]$$

$$+K_{+1,PDE+CaM::Ca4}[PDE][CaM::Ca_4] - K_{-1,PDE+CaM::Ca4}[PDE_{active}]$$

$$\frac{d[PDE_{active}::cGMP]}{dt} = K_{+1,PDEactive}[PDE_{active}][cGMP] - K_{-1,PDEactive}[PDE_{active}::cGMP]$$

$$-K_{+2,PDEactive}[PDE_{active}::cGMP]$$

Membrane current

$$\frac{dV_{Membrane}}{dt} = \frac{1}{C} \bullet I_{Total} = \frac{1}{C} \bullet (I_{CNG} + I_{VG})$$

$$I_{CNG} = I_{CNG,max}\left(\frac{[cGMP]^{n_{CNG}}}{EC50_{CNG}^{n_{CNG}} + [cGMP]^{n_{CNG}}}\right)(V_{Membrane} - V_{R,CNG})$$

$$I_{VG} = Ef_{VG}\{6.0e^{-5}V_{Membrane}^{3} + 8.1e^{-3}V_{Membrane}^{2} + 0.487V_{Membrane} + 18.2\}$$

Normalized GCaMP fluorescence

$$\text{Normalized Fluorescence of G-CaMP} = \frac{\left[Ca^{2+}\right]^{3.3}}{0.235^{3.3}+\left[Ca^{2+}\right]^{3.3}}$$
